# Ethyl Vanillin Rapid Crystallization from Carboxymethyl Chitosan Ion-Switchable Hydrogels

**DOI:** 10.3390/gels9040335

**Published:** 2023-04-14

**Authors:** Chenghong Huang, Hong Tang, Xiaorong Huang, Hongjie Chen, Kang Yang, Qi Yin, Lin Zhang, Xia Li, Xue Mou, Shuangkou Chen, Yuchan Zhang, Yan Hu

**Affiliations:** 1 School of Chemistry and Chemical Engineering, Chongqing University of Science and Technology, Chongqing 401331, China; 20202050591@cqust.edu.cn (H.T.); 2021205143@cqust.edu.cn (X.H.); 2021205202@cqust.edu.cn (H.C.); 2022205037@cqust.edu.cn (K.Y.); 2022205021@cqust.edu.cn (Q.Y.); 2022205044@cqust.edu.cn (L.Z.); lixia@cqust.edu.cn (X.L.); 2020446480@cqust.edu.cn (X.M.); 2013008@cqust.edu.cn (S.C.); 2 Institute of Life Science, And Laboratory of Tissue and Cell Biology, Lab Teaching & Management Center, Chongqing Medical University, Chongqing 400016, China; zhangyc@cqmu.edu.cn; 3 Tuberculosis Reference Laboratory, Chongqing Tuberculosis Control Institute, Chongqing 400050, China; huyanz025@163.com

**Keywords:** EVA, CMCS, crystallization, nanoconfinement

## Abstract

Polymer gels are usually used for crystal growth as the recovered crystals have better properties. Fast crystallization under nanoscale confinement holds great benefits, especially in polymer microgels as its tunable microstructures. This study demonstrated that ethyl vanillin can be quickly crystallized from carboxymethyl chitosan/ethyl vanillin co-mixture gels via classical swift cooling method and supersaturation. It found that EVA appeared with bulk filament crystals accelerated by a large quantity of nanoconfinement microregions resulted from space-formatted hydrogen network between EVA and CMCS when their concentration exceeds 1:1.4 and may occasionally arise when the concentration less than 1:0.8. It was observed that EVA crystal growth has two models involving hang-wall growth at the air-liquid interface at the contact line, as well as extrude-bubble growth at any sites on the liquid surface. Further investigations found that EVA crystals can be recovered from as-prepared ion-switchable CMCS gels by 0.1 M hydrochloric acid or acetic acid without defects. Consequently, the proposed method may offer an available scheme for a large-scale preparation of API analogs.

## 1. Introduction

Vanillin (4-hydroxy-3-methoxy benzaldehyde, Van) and ethyl vanillin (EVA) belong to aroma chemicals obtained from Kraft lignin [1], microorganism fermentation [2] or chemical synthesis [3], which are in-demand largely because they can be used as food additives [4], antimicrobial [5], anti-inflammation [6], and anti-cancer [7] drugs, or as optical material [8]. As early as 1991, Singh et al. [9] found that Van/EVA crystals were polymorphic with conventional type I and type Ⅱ crystallized from chloroform-carbon tetrachloride solution but type Ⅲ and type Ⅳ only discovered from molten state. Subsequently, many researchers have developed various techniques to grow Van or EVA crystal in water [10], methanol and ethanol [11], methanol-chloroform co-solvent [8], oil-extrusion method [12], nickel foam template [13] and vapor diffusion crystallization [14]. These methods are effective and suitable for use but involve inevitable shortcomings of long duration, using organic solvents, of being low quality, or needing sophisticated equipment. Thus, it is essential to develop a facile method with merits of easy operation and without organic solvents usage to prepare EVA crystals for large-scale application.

Polymer gels based on polymers are usually used for crystal growth as the recovered crystals have better properties [15]. Furthermore, polymer gels with tunable microstructure [16,17] can control nucleation and polymorphism through formation of local microregions with nanoconfinement effect [18]. Thus, the gel is thought to act as an inert matrix and may even be cracked because it is a reversible process if formed chemical structure are ion-switchable, which can be destroyed by many factors, such as temperature, pH, sonication and ions. Therefore, the reversible nature of designed polymer gels can be used to recover crystals easily [19,20]. Polymers can also affect the crystal phase and state during formation of hydrate [21,22] or selective crystallization of polymorphs with tunable microstructure induced effects of nanoconfinement and interfacial interactions [23,24]. These characteristics enable the wide application of polymer gels in screening of drug active ingredients in pharmaceutical industry. Recently, ion-switchable polymer gel especially with self-healing function gels [25], can be effectively used for active pharmaceutical ingredient (API) separation [26]. Van/EVA molecules have an active aldehyde group and is able to form a Schiff base structure with a polymer [27] containing amino groups with an outstanding self-healing function [28]. It can be assumed that the recovery of EVA can be achieved by controlling the reaction condition so that only reversible hydrogen bonds are formed, but the Schiff base reaction will not happen.

**Scheme 1:**
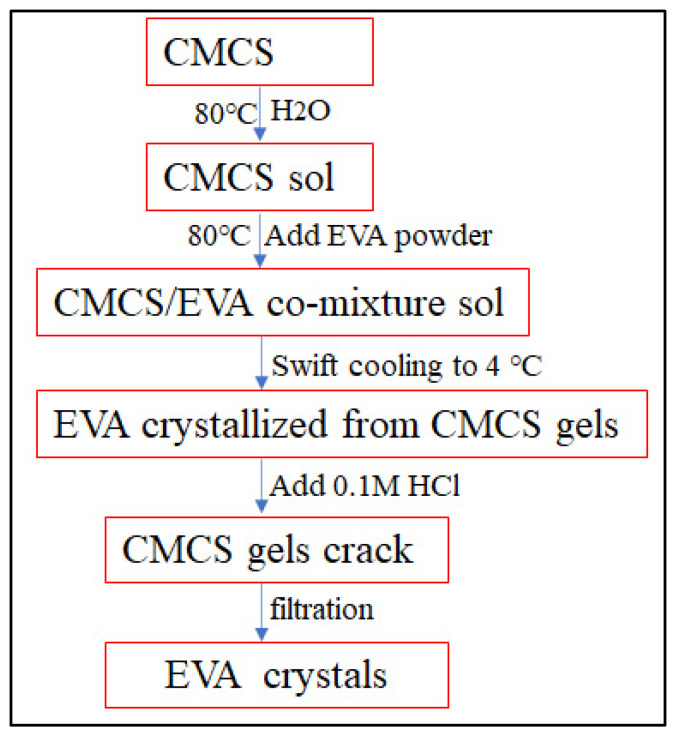
Process chart of EVA crystallization from CMCS gels.

Thereafter, we present a facile method to recover EVA crystals from carboxymethyl chitosan (CMCS) gels through method of swift cooling and supersaturation. The basic principle is explained in Scheme 1. A certain quantity of EVA was dissolved first in CMCS hot solution and then quickly crystallized from the as-prepared co-mixture sol by swift supersaturation to form supersaturation as a result of nanoconfinement effect [29] from space-formatted hydrogen bond networks between CMCS and EVA exclusion of thought to undergo a Schiff base reaction. The latter can be efficiently recovered from the ion-switchable CMCS gel using hydrochloric acid or acetic acid with perfect quality.

## 2. Results and Discussions

### 2.1. Effect of CMCS Molecular Weight on Gel Formation

Chitosan (CS) is a repetitive unit polymer of cationic (1-4)-2-amino-2-deoxy-β-D-glucan. CS has the distinctive feature of carrying predominant units of amino groups, making it wear more cationic charges. Such groups can behave as cationic in acidic media, leading to it often display polyelectrolyte behavior in solution [30]. Carboxymethyl chitosan (CMCS) is an important CS derivative. Our prepared CMCS [31,32] powder is soluble both in acidic and in alkaline aqueous environments, as the carboxymethylation reaction of CS principally occurred hydroxyl moieties as a result of degree of functionalization is nearly higher than 50%. According to our determination, the degree of carboxyl substitution of CMCS in this study is 1.029, with 6% of the substitutions occurring at the C_2_-NH_2_ position and the rest at the C_6_-OH position. The prepared CMCS were stored at room temperature.

The molecular weight effect of the CMCS gel-forming was first systematically investigated. Firstly,0.5 g CMCS with MW of 2 × 10^3^, 10 × 10^4^ and 15 × 10^4^ were separately co-dissolved with 0.5 g EVA to prepare co-mixture solution. We found that CMCS with 2 × 10^3^ MW can only form relative viscosity sol, but ones with 2 × 10^4^ and 15 × 10^4^ can come into being as elastic gels under low temperatures (lower than normal temperatures). This can be expressed as, the higher the molecular weight, the harder the gel hardness. It was reckoned that small molecule CMCS are inclined to form micelle in water solution with better fluidity. However, larger CMCS molecules are inclined to develop hydrogen bond networks as the intramolecular carboxyl group will interact with EVA, in which water molecules will be tightly maintained and are very helpful for gel formation. Many factors, including pH value, temperature, reagents, and degree of chemical substitution can significantly affect its solubility. Both prepared CMCS sol or gel will be destroyed by heating and acid environment. The change is a reversible process, indicating that the chemical structure of CMCS is dominated by forming secondary bonds. In contrast, higher MW CMCS can develop higher hardness gels that can tolerate higher temperature. This feature helps EVA to form more powerful tightening forces during crystallization process. Thus, CMCS with 15 × 10^4^ MW was rationally selected in later work. Further investigations demonstrated that cooling rate also obviously impact the gel formation, that is, the swifter the cooling rate, the quicker the gel formation. For example, the naked-eye EVA crystallization from hot co-mixture gels will soon appear within one hour by placing it to 4 ℃ refrigerator, yet it takes at least fifteen hours to be appear when placed at room temperature, as the side chains of CMCS will contribute to the formation of strong chemical forces through the formation of local bonding regions when temperatures decrease.

### 2.2. EVA Crystallization in CMCS Gels

**Figure 1:**
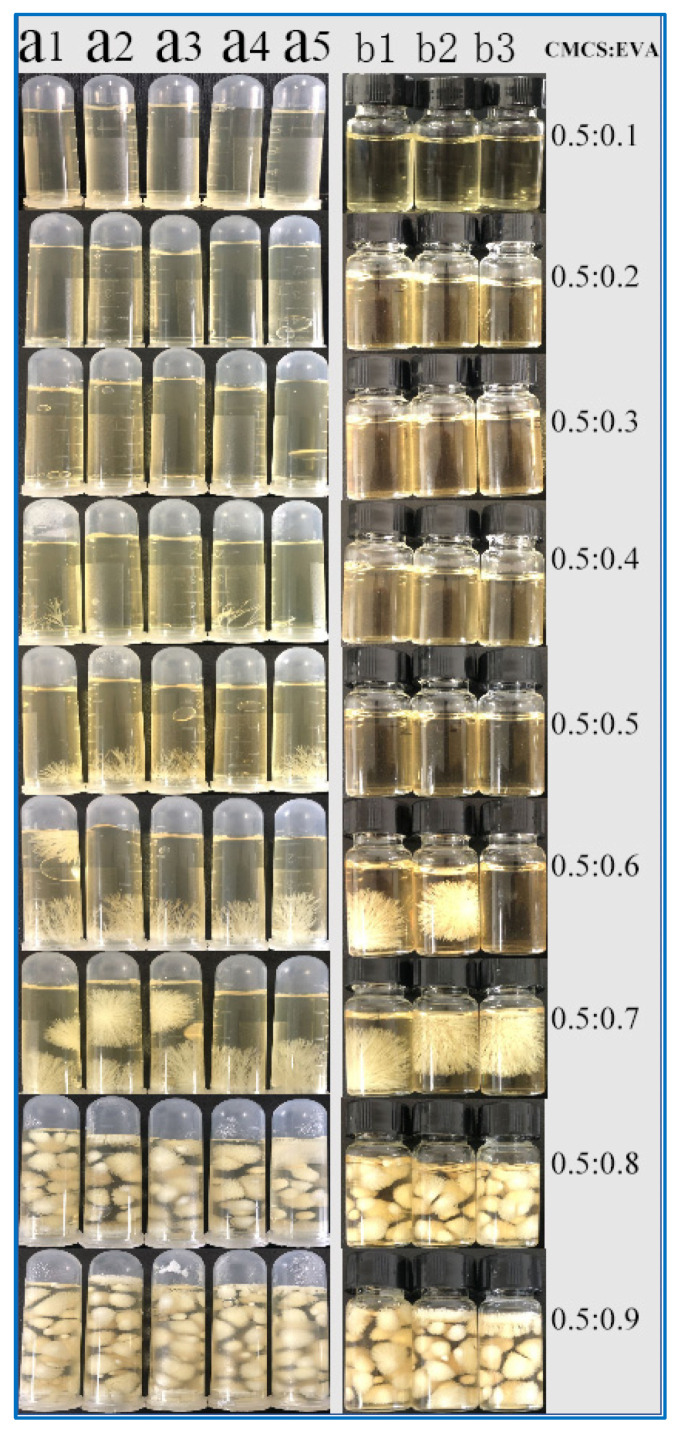
EVA crystallization from CMCS gels with different concentration ratio (**a1**–**a5** and **b1**–**b3**) in centrifuge tube (five repeats **a1**–**a5**) and ampoule bottle (three repeats **b1**–**b3**).

To explore the crystallization law of EVA in CMCS gels, we use fixed CMCS mass concentration and change the quantity of EVA to conduct the crystallization study. Consistent with the classical nucleation theory [33], supersaturation concentration is considered to be prerequisite for EVA crystallization in CMCS gels. EVA will rapidly crystallize (in less than 30 min) in CMCS/EVA co-mixture gels during the transition process from sol to gel by swift cooling, whether in a centrifuge tube or an ampoule bottle (Figure 1). The difference is that EVA crystallization in the former will achieve under the condition of concentration ratio exceeding 1:0.8, while in the latter it will reach up to 1:1.2, which is caused by the different pressure of supersaturation caused by adjusting the tightness of the rotary lid. We also found that EVA crystals from medium concentration (centrifuge tube at 1:0.8–1:1.4, ampoule bottle at 1:1.2–1:1.4) are scattered growth, resulting from less mobility within sparse CMCS sol, but are relatively packed into local micelles at higher concentrations (mass ratio ˃ 1:1.4), because EVA will self-aggregate to assemble a regular molecular arrangement. This is very conducive to supersaturation by the formation of an inter-molecular hydrogen bonding network [34], owing to the reduction of the mobility of sol with decreases of temperature. The growth of EVA in macromolecular CMCS gel is obviously faster than that in low molecular CMCS gels, because the former has more hydrogen bonding networks with swift cooling and gel formation.

**Figure 2:**
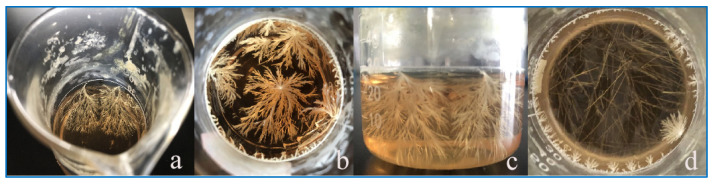
EVA hang-wall growth (**a**) at air/liquid interface or bulge growth (**b**,**c**) on liquid surface, and occasionally acquired linear EVA crystals (**d**) in CMCS/EVA con-mixture gels.

Crystallization is an important issue in the pharmaceutical industry. The quality of drugs is usually affected by the content, solubility, stability, reactivity, and even polymorphism of API [35]. Although many preparation methods of Van crystals have been listed above [10,12,14,36], industrial large-scale preparation from solution is preferred because the acquired crystals are high quality and easy to recover. Crystals from solution usually start from formation a supersaturated solution concentration. Recently, research reported that solution crystallization is initiated at the edge of a drying drop where the solution is evaporated [11], or the crystal particles are rearranged at the air-liquid solid interface where colloidal suspensions occur [37]. These understandings were further confirmed by Capes’s [38] experiment, which investigated the selective crystallization of paracetamol polymorphism, in which paracetamol will preferentially crystallize at the contact line of the evaporating solution. In this study, EVA crystallization depends on supersaturation theory, as illustrated by beaker growth (Figure 2). It can be seen that EVA crystals will easily emerge on the contact line at the air/liquid interface by water evaporation. With the liquid level’s decline, supersaturation will occur, which is named hang-wall growth (Figure 2a) due to an endowed higher concentration of EVA, or bulge growth (Figure 2b,c) at any position mediated by a randomly formed crystal nucleus on the liquid surface when EVA specific micelles are formed during gel process, probably making it as a random event. Subsequently, the EVA crystal will transform into branch growth along the top to the bottom in the beaker and later turn into a tree-shape (Figure 2c) [23]. The EVA crystals in the beaker distribution are without direction selectivity. Many linear EVA crystals (Figure 2d) are accidentally acquired and thereupon switch into tree growth within 2 h. Whether the flash in the pan linear Van relates to polymorphism will be reported in future studies.

**Figure 3:**
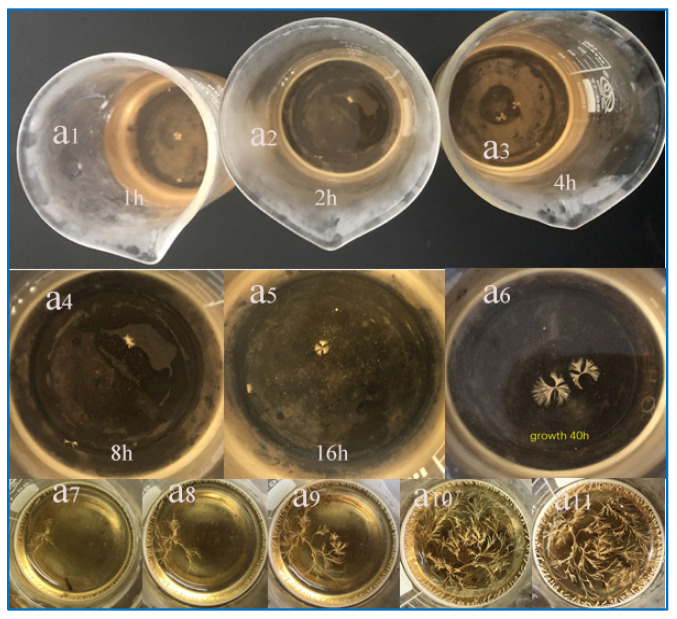
EVA crystal growth in CMCS/EVA co-mixture gels with appearances from leaves (**a1–a6**) to trees ramification (**a7–a11**).

The sol-gel method is a common and effective method to study gel formation with the change from sol to gel. Still further, sol-gel method can be availably utilized to prepare molecular imprinted polymers (MIPs), as the obtained MIPs provide outstanding chemical inertness, physical rigidity and hydrophilic properties [39]. In the transition from sol to gel, chemical bonds from secondary to covalent bonds also occur. For the purposes of this experiment, early (within 1 h) naked-eye visible EVA crystals in CMCS/EVA co-mixture gels exhibited three-, four- or six-leaved appearances (section view). After that, they grew into tree-like ramification growths (Figure 3a1–a9). The crystal growth is a gradual process. It is obvious that the crystal growth of EVA is accompanied by the coming into being of gels. After sixteen hours, crystals extended into the interior of gels and developed into plenty of branches with an unrestrained stretching growth model. As time goes by, EVA crystals will penetrate the CMCS gels in the beaker whatever on the wall or at the middle of any sites with the conversion process from sol to gel, and then form flower pattern growth (Figure 3a7–a11). EVA crystals are difficult to separate from CMCS gels without a broken agent addition.

### 2.3. Crystallization Law and Mechanism of EVA in CMCS Gel

EVA containing hydrophobic benzene rings tend to aggregate into nucleate in solution [40] because of the water molecular exclusion effect. The solubility of EVA in water is related to the temperature. Swift cooling is suitable for EVA crystallization [41]. EVA molecular aggregates comply with spatial orientation according to the principle of minimum energy. Crystal properties related to polymorphism, crystal morphology and particle-size distribution are usually determined by crystal nucleation and later development. Teknova et al. [42], by using dynamic light scintillation technology (DLS), determined the vanillin (Van) crystal nucleus during its growth process and found that Van will develop into clusters by crystal particle collision-cooling, and finally cultivate into millimetre size, even naked-eye visible, crystals, or alternatively develop unstable an amorphous phase or anterior crystal structure. Sundareswaran et al. [13] found that solute concentration-mediated supersaturation can trigger crystal nucleation and polymorphism by varied solute concentration. Sometimes nucleation and metastable region coexist. Lower supersaturation is more helpful for I and moderate supersaturation more conducive to II Nucleation. Higher supersaturation is only in favor of II Nucleation. The crystals may be switchable by any factors at-most optimized conditions.

**Figure 4:**
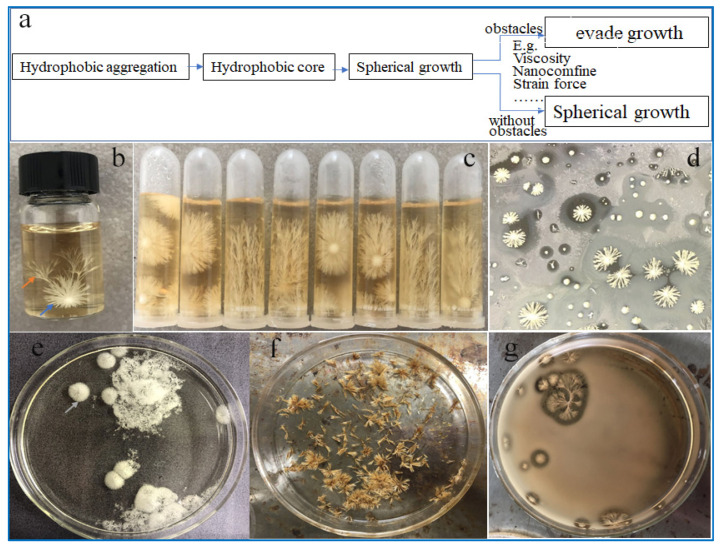
EVA in CMCS sol. (**a**) growth pattern of EVA crystal in CMCS gels, (**b**) Free spherical growth in sol (blue arrow) or tree growth in CMCS gels (yellow arrow)), (**c**) Hydrophobic aggregation core of EVA in CMCS sol, (**d**) spherical aggregation of CMCS/EVA on silicon wafer surface; (**e**) 2 × 10^3^ molecular weight CMCS forms loose velvet flower in CMCS sol (grey arrow); (**f**) dried state of velvet flower, (**g**) 15 × 10^4^ molecular weight CMCS forms CMCS/EVA tree crystals in plate.

The presumptive crystallization mechanism is illustrated as Figure 4a. Further observations disclose that EVA will develop a spherical growth in limited space on the basis of molecule configuration beneficial to lower energy. (Figure 4b, blue arrow denoted). On the other hand, it leads to pursuing growth like of plant budding if it is encountered with nanoconfinement, gel stress and evaporation contraction force [43]. We dispatched the CMCS/EVA hot co-mixture solution into eight centrifuge tubes and a typical hydrophobic condensed nucleus was unexceptionally formed (Figure 4c). The nucleus then uniformly grew in all directions, maintaining a consistent size about 0.5 cm in diameter. More favorable verification for the assumed hypothesis is from experiments in which EVA condensed a nucleus present in spherical growth after water evaporation when CMCS/EVA hot co-mixture was uniformly spread on a silicon surface in order to reduce strain of gel formation (Figure 4d) and shows velvet flower growth on the premise of exclusion of forming hydrogen bond network when 2 × 10^3^ MW CMCS was used (Figure 4e). The velvet structure is very fragile and its structure will be destroyed by gently shaking. Dried CMCS/EVA composites finally came into being with a spherulite shape (Figure 4f), attributed to the self-aggregation of EVA by water driving, which is basically in accordance with an early report from E. Gomes’ research [1] and demonstrated by Guella’s research [44]. Nevertheless, EVA crystals can be gradually formed at individual sites when CMCS/EVA (MW of CMCS higher than 2 × 10^4^) hot co-mixture sol is dumped into plate with slow evaporation maintaining 1 cm thickness (Figure 4g).

**Figure 5:**
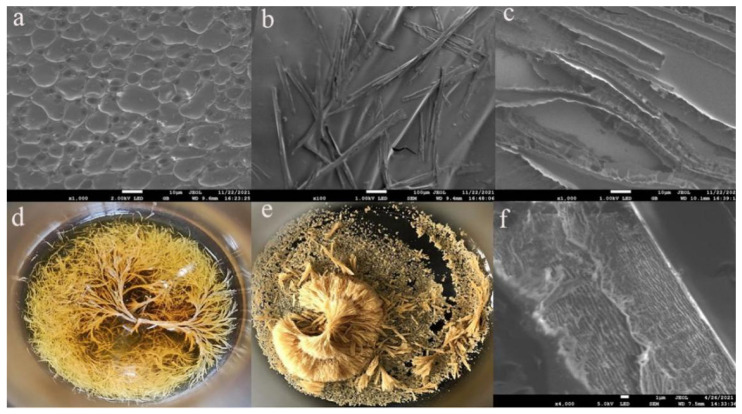
SEM images of dried CMCS/EVA with at 2 × 10^3^ (**a**), 2 × 10^4^ (**b**) and (**c**) 15 × 10^4^ molecular weight, which are stirred in a magnetic mixer at 120 rpm for 15 days; (**d**) EVA precipitated with symmetrical shapes (**e**) dried d and (**f**) regular structure of e.

Micelle structures for CMCS/EVA (MW of CMCS is 2 × 10^3^) in sol be further confirmed by SEM images. From Figure 5a it can be seen that CMCS/EVA behaves as a micelle structure with uniform vesicles. Nevertheless, a hinge-tangled structure (Figure 5b) of CMCS using MW 2 × 10^4^ and a longer helical structure (Figure 5c) with 2× 10^5^ MW were be observed as the torsional properties can significantly reduce the modulus of CMCS [45] and largely lock the group sites under gel strain, which is helpful for molecular self-assembling [46]. The former will be precipitated into a treetop pattern (Figure 5d) and ultimately dried in battlements with a tailor-made appearance [47] (Figure 5e), like the observed microstructures in Figure 5e, which is especially consistent with the reported result [48].

**Figure 6:**
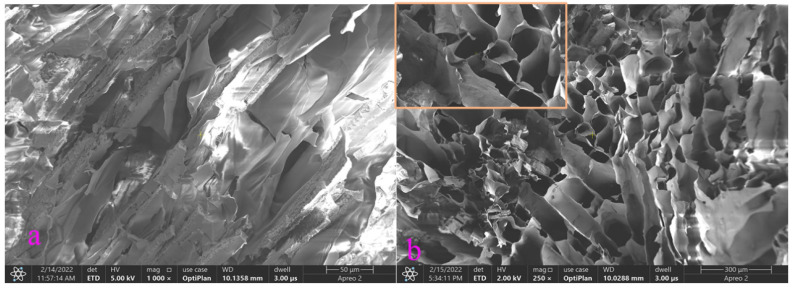
Irregular arrangement of CMCS blank gels (**a**) and regular arrangement of CMCS/EVA co-mixture gels (**b**) in SEM images.

EVA can theoretically cross-link with CMCS through a Schiff base structure, which usually requires a long reaction time at 60 °C, and the chemical linkage is often reckoned as being hydrolyzed without a reducing agent. On the other hand, extensive a hydrogen bond network (see Figure S1) can be formed between an amino-group of EVA and a carboxyl group of CMCS, which is the chemical structural basis of transformation from sol to gel. We speculate that the crystallization of EVA in the hydrogen bond network will lead to the formation of a homogeneous small hydrogen bond compartment. The optimized reaction condition of 80 °C for 5 min reaction, as well as swift cooling, easily achieve formation supersaturation, meanwhile avoiding formation of a Schiff base. Results of SEM in Figure 6a revealed an irregular arrangement for blank CMCS gels (MW 15 × 10^4^), while a regular arrangement for CMCS/EVA co-mixture gels microstructure. CMCS normally takes a similar conformation of helical structure with CS [49]. In aqueous solution, the carboxyl and amino groups of CMCS disassociate to form a charge and are therefore greatly influenced by the pH environment.

EVA crystallization is accompanied the CMCS gel process, and vice versa, it also quickened the formation of a hydrogen bond network. Therefore, a large number of small space compartments (Figure 6b) formatted with nanoconfinement effect accompanied this process. EVA immediately crystallized within such confinement regions by a local supersaturation effect [50]. EVA can easily fall off from these small compartments in the same way as sand falling, so far as to be visible by a common magnifier. Interestingly, granular crystals were obtained after freeze-drying, while bulk crystals were obtained after acid lysis. This indicates that hydrogen bonds play an important role in the crystal formation of EVA.

### 2.4. Recovery and Characterization

**Figure 7:**
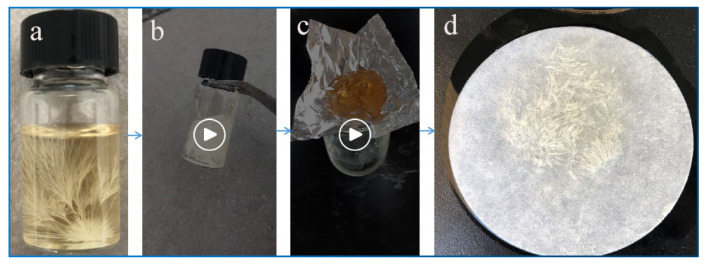
EVA crystals were recovered from CMCS/EVA co-mixture gels. (**a**) EVA growth in CMCS gel, (**b**) 0.1M HCl hydrolyze CMCS gels, (**c**) EVA crystals float in CMCS solution and (**d**) EVA crystals recovered from membrane filtration.

Further investigations demonstrated that CMCS/EVA co-mixture gels can be hydrolyzed by dilute hydrochloric acid or acetic acid. Practical significance of such ion-switchable gel is that it can largely prepare a similar template to produce many active pharmaceutical ingredients (API). The detailed illustration is taken from a video of Figure S2 and Figure 7.

**Figure 8:**
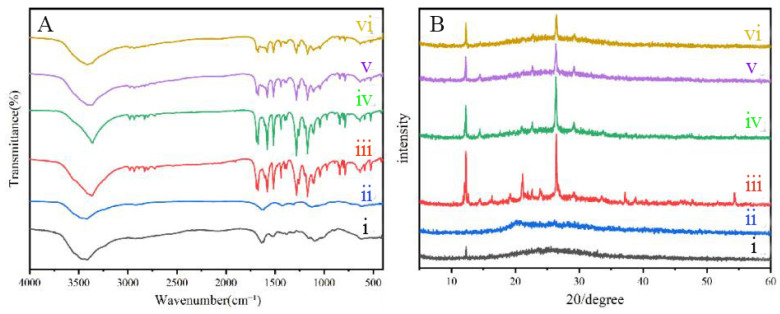
FT-IR (**A**) and XRD (**B**) of LMW CMCS (ⅰ), CMCS (ⅱ), EVA (ⅲ), CMCS/EVA dried gel (ⅳ), EVA crystals (ⅴ, 25 °C obtained) and EVA crystals (ⅵ, 4 ℃ obtained). Detailed descriptions please see the text.

Figure 8 shows FT-IR (A) and XRD (B) results of raw materials and obtained EVA crystals at different temperatures. Figure 8A shows the FT-IR results of LMW CMCS, CMCS, EVA, and CMCS/EVA dried gel, respectively. In this, 3370 cm^−1^ and 2933 cm^−1^ can be separately attributed to stretching vibration peaks of –OH and –CH of aromatic ring. The stretching vibration peaks of 2980 cm^−1^, 2828 cm^−1^ and 2720 cm^−1^ can be assigned to C–H groups of –CHO, the stretching vibration peaks of 1686 cm^−1^ can be endowed to C=O groups. Additionally, strong peaks of 1512 cm^−1^ should be attributed to stretching vibration, and in-plane deformation vibration of C–H of –CHO, 1438 cm^−1^ can be appointed to stretching vibration peaks of C–O of Ar–OH, 1276 cm^−1^ whilst 104 0 cm^−1^ can be specified as stretching the vibration peaks of C–O of the oxyethyl group; 840 cm^−1^, 800 cm^−1^ and 780 cm^−1^ are designated to replace absorption peaks. Our previous research [32] demonstrated that the amide band (1616 cm^−1^) exists in FT-IR after EVA is successfully incorporated into CMCS if the Schiff base bonds are achieved. Nevertheless, no vibration peaks of imide linkage were found in the current FT-IR image for dried CMCS/EVA gels, which illustrates that only hydrogen bonds interactions occurred between EVA and CMCS. Figure 8B shows corresponding XRD results, which are consistent with the EVA characteristic peaks from 2θ = 13° to 40°, while the positions of the sample diffraction characteristic peaks mostly overlapped.

Faultless EVA crystals can be easily recovered by cellulose paper filtration after CMCS/EVA co-mixture gel is hydrolyzed by 0.1 M hydrochloric acid or acetic acid. The acid lysis velocity is positively correlated with the gel breaking velocity. However, higher acidity will also affect the quality of EVA crystals to some extent, so 0.1M hydrochloric acid was finally taken as the working concentration, besides economic and environmental considerations. Both the structure and quality of EVA are perfect compared with native EVA powder, as verified by FT-IR and XRD characterization. The recovery rate can reach up to 95% by our calculation. The proposed facile method based on ion-switchable CMCS gel together with nanoconfinement effect and supersaturation takes advantage of bring rapid, environment-friendly and highly efficient for EVA preparation.

## 3. Conclusions

Polymer gels with adjustable structure based on polymers are usually used for crystal growth as the recovered crystals have better properties. Ion-switchable gel, e.g., CMCS, is a powerful tool for preparing high-quality crystals. Soluble EVA can rapidly crystalize from CMCS/EVA co-mixture gel with a filamentary shape due to swift cooling. Results demonstrated EVA crystallization will only happen when the concentration ratio exceeds 1:0.8 in centrifuge tubes or 1:1.2 in ampoule bottles. Induction time of crystallization depends on the cooling rate. Investigations found that EVA will grow by the hang-wall model at the air-liquid contact line interface, or by extrude-bubble growth at any site on the liquid surface in a beaker by supersaturation formation. Rapid EVA crystallization was interpreted as the introduction of space-formatted compartments with a nanoscale confinement effect from hydrogen bond networks, and restricted molecular assembly under CMCS chain strain exclusion of Schiff base formation within a short-duration chemical reaction. The quality of the EVA crystal obtained depends on the cooling velocity. The proposed method carries advantages of being rapid, environmentally friendly and easy to operate. It is an effective method for drug API preparation in the preparation industry.

## 4. Experimental

### 4.1. Reagents

Carboxymethyl chitosan (100 kDa–150 kDa, 90% deacetylation) and ethyl vanillin were purchased from Aladdin biochemical reagents; low molecule weight CMCS (LMW CMCS) with MW of 2 × 10^3^, 10 × 10^4^ and 15 × 10^4^ were prepared according to previous work [30]. Silicon wafer were bought from the China Institute of Nonferrous Metals. Hydrochloric acid was from Chongqing Chuandong Chemical Co., Ltd. Chongqing, China. All the other reagents were analytically pure. Deionized water (18MX.cm) was made by our laboratory-equipped molar water machine.

### 4.2. Apparatus

Centrifuge (TG16-W, Changsha Xiangyi Centrifuge Instrument Co., Ltd.Changsha, China), Constant temperature water bath (HH-S, Jintan Zhengji Instrument Co., Ltd.Zhejiang, China), Fourier-infrared spectrometer (Thermo Nicolet), X-ray crystal meter (Shimadzu, XRD-70000) and scanning electron microscope (JEM-2100F, JEOL) were supplied by Chongqing University of Science and Technology.

### 4.3. CMCS/EVA Co-Mixture Gel Preparation by Sol-Gel Method

A certain amount of CMCS powder was dissolved in deionized water, and 100 rpm magnetic agitated till it completely dissolved at 60 °C. Then, we heated up the CMCS solution to 80 °C. An additional appropriate amount of EVA powder was stepwise added to the as-prepared CMCS solution to form co-mixed sol for 5 min with continuous agitation. At last, we transferred the sol to a centrifuge tube or ampoule and promptly placed it into a 4 °C refrigerator for EVA crystallization growth, along with the development of CMCS gels.

### 4.4. EVA Nucleation and Rapid Crystallization

In order to comprehend the detailed nucleation and crystallization mechanism of EVA from CMCS gels, the CMCS/EVA co-mixture sols were respectively delivered to centrifuge tube, ampoule bottle and silicon wafer surface. After the gels formed, or water gradually evaporated, EVA crystals crystallized. We recorded the induction time of EVA crystals and identified its characteristics.

### 4.5. Crystal Recover and Calculation

When CMCS/EVA has crystallized and aged, appropriate 0.1M HCL was added into the co-mixture gels. After the CMCS gels thoroughly dissociated, EVA crystals were floated in solution.Then weigh and recover the EVA crystals. Recovery rate will be accordingly calculated by the following formula:

(1) rate of recovery=recovered EVA weight EVA raw weight×100%

### 4.6. Characterization

(1) Fourier transform-infrared spectroscopy(FT-IR). The samples were mixed with KBr(m/m = 1:10) and delivered for tablet compressing. The Nicolet 10 (Thermo Nicolet) was used for measurement. The scanning wavelength is from 4000 to 400 cm^−1^. (2) X-ray diffraction (XRD). Crystal structures were determined by a X-ray diffraction analyzer (Shimadzu, Japan XRD-70000). The 2θ scanning range is 5~60°, the speed is 5°/min. (3) Scanning Electron Microscope (SEM) observation. All samples has lyophilization performed for at least 24 h. SEM images of the prepared materials were taken on a JEOL JEM 2010 microscope operating at 200 kV. (5) The MW and degree of dissociation(DDs) of different CMCS were determined by reported method of Lee et al. [51].

## Data Availability

Not applicable.

## Supplementary Materials

The following are available online at https://www.mdpi.com/article/10.3390/gels9040335/s1, Figure S1: Hydrogen bond network formed between EVA and CMCS by molecular simulation, Video S1: EVA crystal were recovered from CMCS gels by acidolysis.

## References

[1] Gomes E., Rodrigues A. (2020). Crystallization of vanillin from kraft lignin oxidation. Sep. Purif. Technol..

[2] Chakraborty D., Gupta G., Kaur B. (2016). Metabolic engineering of E. coli top 10 for production of vanillin through FA catabolic pathway and bioprocess optimization using RSM. Protein Expr. Purif..

[3] Augugliaro V., Camera-Roda G., Loddo V., Palmisano G., Palmisano L., Parrino F., Puma M.A. (2012). Synthesis of vanillin in water by TiO_2_ photocatalysis. Appl. Catal. B Environ..

[4] Amouheydari M., Ehsani M.R., Javadi I. (2020). Effect of a dietary supplement composed of hydrolyzed milk proteins and vanillin on the reduction of infection and oxidative stress induced by chemotherapy. J. Food Biochem..

[5] Fayeulle A., Trudel E., Damiens A., Josse A., Youssef N.B.H., Vigneron P., Vayssade M., Rossi C., Ceballos C. (2021). Antimicrobial and antioxidant activities of amines derived from vanillin as potential preservatives: Impact of the substituent chain length and polarity. Sustain. Chem. Pharm..

[6] Jung H.-J., Song Y.S., Kim K., Lim C.-J., Park E.-H. (2010). Assessment of the anti-angiogenic, anti-inflammatory and antino-ciceptive properties of ethyl vanillin. Arch. Pharmacal Res..

[7] Li G., Kong B., Tong Q., Li Y., Chen L., Zeng J., Yu H., Xie X., Zhang J. (2021). Vanillin downregulates NNMT and attenuates NNMT-related resistance to 5-fluorouracil via ROS-induced cell apoptosis in colorectal cancer cells. Oncol. Rep..

[8] Singh O., Singh N. (2001). Growth of vanillin crystals for second harmonic generation (SHG) applications in the near-IR wavelength region. J. Cryst. Growth.

[9] McCrone W.C. (1950). Crystallographic data Vanillin I (3-Methoxy-4-hydroxybenzaldehyde. Anal. Chem..

[10] Sundareswaran S., Karuppannan S. (2021). Nucleation control and separation of vanillin polymorphs I and II through the swift cooling crystallization process. Crystengcomm.

[11] Singh N., Henningsen T., Metz E., Hamacher R., Cumberledge E., Hopkins R., Mazelsky R. (1991). Solution growth of vanillin single crystals. Mater. Lett..

[12] Friberg S.E., Heisig C.C., Kayali I. (1996). Precipitation of Vanillin from a Water/Soybean Oil Emulsion Stabilized by Lecithin. Flavour Fragr. J..

[13] Sundareswaran S., Karuppannan S. (2020). Supersaturation Dependent Separation of Vanillin Polymorphs from Aqueous Solution in the Presence of Ni-Foam as Template. Cryst. Res. Technol..

[14] Supriya S., Sushmitha S., Srinivasan K. (2019). Effective Control of Liquid-Liquid Phase Separation and Nucleation of Vanillin Single crystals through Vapour Diffusion Crystallization Process at selected solvent environments. Cryst. Growth Des..

[15] Lloyd G., Steed J. (2009). Anion-tuning of supramolecular gel properties. Nat. Chem..

[16] Diao Y., Helgeson M.E., Myerson A.S., Hatton T.A., Doyle P.S., Trout B.L. (2011). Controlled Nucleation from Solution Using Polymer Microgels. J. Am. Chem. Soc..

[17] Safari M., Boigues L.L., Shi G., Maiz J., Liu G., Wang D., Mijangos C., Müller A.J. (2020). Effect of Nanoconfinement on the Iso-dimorphic Crystallization of Poly(butylene succinate-ran-caprolactone) Random Copolymers. Macromolecules.

[18] Jiang Q., Ward M.D. (2013). Crystallization under nanoscale confinement. Chem. Soc. Rev..

[19] Parker A.S., Taylor L.S., Beaudoin S.P. (2021). Polymer effects on crystallization at the amorphous atazanavir-water interface. J. Cryst. Growth.

[20] Zhou D., Xu M., Tan R., Sun Y., Ma Z., Li J., Dong X.-H. (2021). Quantitatively unravel the effect of chain length heterogeneity on polymer crystallization using discrete oligo l-lactide. Polymer.

[21] Tian F., Baldursdottir S.G., Rantanen J. (2008). Effects of Polymer Additives on the Crystallization of Hydrates: A Molecular-Level Modulation. Mol. Pharm..

[22] Sudha C., Nandhini R., Srinivasan K. (2014). Polymer-Induced Selective Nucleation of Mono or Ortho Polymorphs of Paracetamol through Swift Cooling of Boiled Aqueous Solution. Cryst. Growth Des..

[23] Diao Y., Whaley K.E., Helgeson M.E., Woldeyes M.A., Doyle P.S., Myerson A.S., Hatton T.A., Trout B.L. (2011). Gel-Induced Selective Crystallization of Polymorphs. J. Am. Chem. Soc..

[24] Mohammadi R.S., Zolali A.M., Tabatabaei S.H., Ajji A. (2020). Nanoconfinement Induced Direct Formation of Form I and III Crystals inside in Situ Formed Poly(butene-1) Nanofibrils. Macromolecules.

[25] Li C.-H., Wang C., Keplinger C., Zuo J.-L., Jin L., Sun Y., Zheng P., Cao Y., Lissel F., Linder C. (2016). A highly stretchable autonomous self-healing elastomer. Nat. Chem..

[26] Foster J., Piepenbrock M.-O.M., Lloyd G., Clarke N., Howard J.A.K., Steed J. (2010). Anion-switchable supramolecular gels for controlling pharmaceutical crystal growth. Nat. Chem..

[27] Liu Q., Ji N., Xiong L., Sun Q. (2020). Rapid gelling, self-healing, and fluorescence-responsive chitosan hydrogels formed by dy-namic covalent crosslinking. Carbohydr. Polym..

[28] Xu C., Zhan W., Tang X., Mo F., Fu L., Lin B. (2018). Self-healing chitosan/vanillin hydrogels based on Schiff-base bond/hydrogen bond hybrid linkages. Polym. Test..

[29] Beiner M. (2008). Nanoconfinement as a tool to study early stages of polymer crystallization. J. Polym. Sci. Part B Polym. Phys..

[30] Fang Y., Wang K., Li Q., Huang C. (2021). pH responsive release of paclitaxel by self-assembling Chitosan-ethyl vanillin@GNRs nanocomposites. Int. J. Pharm..

[31] Lee K.Y., Ha W.S., Park W.H. (1995). Blood comoatibilitv and biodegradability ofpartially IV-acylated chitosan derivatives. Bio-Mater..

[32] Kong M., Chen X.G., Xing K., Park H.J. (2010). Antimicrobial properties of chitosan and mode of action: A state of the art review. Int. J. Food Microbiol..

[33] Borsagli F.G.M., Mansur A.A., Chagas P., Oliveira L.C., Mansur H.S. (2015). O-carboxymethyl functionalization of chitosan: Complexation and adsorption of Cd (II) and Cr (VI) as heavy metal pollutant ions. React. Funct. Polym..

[34] Pino-García O., Rasmuson Å.C. (2004). Influence of Additives on Nucleation of Vanillin Experiments and Introductory Molecular Simulations. Cryst. Growth Des..

[35] Shen Y., Wang Z., Wang Y., Meng Z., Zhao Z. (2021). A self-healing carboxymethyl chitosan/oxidized carboxymethyl cellulose hydrogel with fluorescent bioprobes for glucose detection. Carbohydr. Polym..

[36] Singhal D., Curatolo W. (2004). Drug polymorphism and dosage form design:a practical perspective. Adv. Drug Deliv. Re-Views.

[37] Deegan R.D., Bakajin O., Dupont T.F., Huber G., Nagel S.R., Witten T.A. (1997). Capillary flow as the cause of ring stains from dried liquid drops. Nature.

[38] Goldenberg L.M., Wagner J., Stumpe J., Paulke B.-R., Görnitz E. (2002). Simple Method for the Preparation of Colloidal Particle Monolayers at the Water/Alkane Interface. Langmuir.

[39] Capes J.S., Cameron R.E. (2006). Contact Line Crystallization To Obtain Metastable Polymorphs. Cryst. Growth Des..

[40] Shang L., Liu C., Watanabe M., Chen B., Hayashi K. (2017). LSPR sensor array based on molecularly imprinted sol-gels for pattern recognition of volatile organic acids. Sensors Actuators B Chem..

[41] Wang K., Tang H., Chen H., Huang X., Fang Y., Men R., Gao J., Wang Y., Wang Y., Huang C. (2022). Synthesis, fabrication and properties research of CS-mPEG-AN nanocomposite. Ferroelectrics.

[42] Wu H., Wang J., Zhou Y., Guo N., Liu Q., Zong S., Bao Y., Hao H. (2017). Solid–liquid phase equilibrium and dissolution properties of ethyl vanillin in pure solvents. J. Chem. Thermodyn..

[43] Sorensen T.J. (2014). Oiling-Out and Crystallization of Vanillin from Aqueous Solutions. Chem. Eng. Technol..

[44] Han M., Rogers S.A., Espinosa-Marzal R.M. (2022). Rheological Characteristics of Ionic Liquids under Nanoconfinement. Langmuir.

[45] Ferrazza R., Rossi B., Guella G. (2014). DOSY-NMR and Raman Investigations on the Self-Aggregation and Cyclodextrin Com-plexation of Vanillin. J. Phys. Chem..

[46] Xu J., Wang S., Wang G.-J.N., Zhu C., Luo S., Jin L., Gu X., Chen S., Feig V.R., To J.W.F. (2017). Highly stretchable polymer semiconductor films through the nanoconfinement effect. Science.

[47] Maor I., Koifman N., Kesselman E., Matsanov P., Shumilin I., Harries D., Weitz I.S. (2021). Molecular self-assembly under nanoconfinement: Indigo carmine scroll structures entrapped within polymeric capsules. Nanoscale.

[48] Huang S., Pang L., Chen Y., Zhou L., Fang S., Yu H. (2018). Hydrogen Bond Induces Hierarchical Self-Assembly in Liq-uid-Crystalline Block Copolymers. Macromol. Rapid Commun..

[49] Nakagawa S., Yoshie N. (2020). Periodic Surface Pattern Induced by Crystallization of Polymer Brushes in Solvents. Macromolecules.

[50] Ogawa K., Yui T., Okuyama K. (2004). Three D structures of chitosan. Int. J. Biological. Macromol..

[51] Taylor M.P. (2017). Polymer Folding in Slitlike Nanoconfinement. Macromolecules.

