# Supplementary figures and images for: Ethyl Vanillin Rapid Crystallization from Carboxymethyl Chitosan Ion-Switchable Hydrogels
Source: Gels. 2023 Apr 14;9(4):335. doi: 10.3390/gels9040335 (PMC10138138; doi:10.3390/gels9040335)

Supplement:

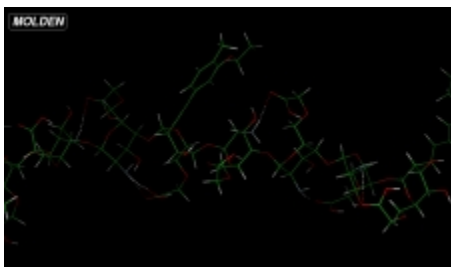

S1 Hydrogen bond network formed between EVA and CMCS by molecular simulation.

Supplement: Supplementary file 1 [file gels-09-00335-s001.zip › gels-2253188-supplementary.pdf]
